# P280: Seroprevalence of measles, rubella, mumps, and varicella among health care workers in Japan

**DOI:** 10.1186/2047-2994-2-S1-P280

**Published:** 2013-06-20

**Authors:** T Watanabe, M Tsuchiya, T Suzuki, T Niwa, H Ohta, N Murakami

**Affiliations:** 1 Infection Control Team, Gifu University Hospital, Gifu, Japan

## Introduction

Immunity to measles, mumps, rubella, and varicella zoster (MMRV) is an important part of infection prevention among health care workers (HCWs), both for their own health and for the health of patients.

## Objectives

To evaluate seroprevalence, antibody levels of HCWs against MMRV were analyzed in an academic medical center in Japan.

## Methods

Serologic screening for MMRV was performed on HCWs in Gifu University Hospital, which carry on measurement of seroprevalence and promote vaccination for eligible HCWs in every 5 years. The specific IgG antibodies were screened quantitatively with use of particle agglutination assay for measles, hemagglutination inhibition assay for rubella, immunosorbent enzyme-linked assay (ELISA) for mumps and varicella. The cut-off value was a titer level of 128 for measles, a titer level of 16 in women and 8 in men for rubella, 6.0 ELISA units/mL for mumps, and 4.0 ELISA units/mL for varicella. In addition, positive conversion ratio was evaluated in HCWs, who were vaccinated in 2007 and measured antibody levels in 2012.

## Results

In total, 1,385 HCWs participated in this survey. Among tested HCWs, 87.6%, 82.4%, 64.2%, and 98.2% had immunity to measles, rubella, mumps, and varicella, respectively. There was no correlation between the seroprevalence of antibodies against MMRV and gender, age, and profession. In a total 129 HCWs who were vaccinated in 2007, the serum antibody levels were measured. Positive conversion ratio against measles, rubella, mumps and varicella were 26.3% (5/19), 59.1% (26/44), 1.9% (1/52), and 64.3% (9/15), respectively.

## Conclusion

The immunity rate to varicella was high among HCWs (98.2%). Meanwhile, the rate to measles, rubella, and mumps was low (64.2-87.6%) in spite of routine screening for the presence of antibodies against MMRV and vaccination in every 5 years. The low positive conversion ratio against measles and mumps might influence the low seroprevalence against those diseases. It is important to carry on examining antibody levels to MMRV among HCWs regularly, to promote vaccination for eligible HCWs, and to consider multiple dose of vaccination.

## Competing interests

None declared

